# The Graduating Classes of 1901

**Published:** 1901-04

**Authors:** 


### THE GRADUATING CLASSES OF 1901.

The period for graduation at the various dental schools having a seven-months' course has arrived, and a large body of young men will pass out into the great world of activities to practise the profession it has cost them three years of time and much outlay of money to secure.

The most important question that can engage the graduate's attention, after location has been secured and a permanent settlement made, is that of association with his fellows in professional work. Any life is proportionately barren if it seeks the cloister of self-isolation. The dental profession is not only an active one, but it demands constant association. Its rapid growth has been largely the result of this associated effort, and no one can fully reach development who fails to become a part of dental organizations.

In connection with this, and hardly second in importance, is the constant and close connection with dental literature. A dentist must be a reader if he would aim to keep step with the almost hourly progress of his calling. He cannot be intelligent without the knowledge thus acquired, for all that he may have absorbed in his college course will soon grow indistinct in his memory unless constantly freshened by new inspirations from periodical literature.

There is another point of equal importance, and that is the cultivation of a moral standard, avoiding those non-ethical things that so frequently mar the truly professional spirit. There is a great need for more of this personal care. It should be manifested in all the relations of active life. Young practitioners should seek those avenues of information that are entirely clear of outside entanglements, and one of these is the truly professional journal.

This number and the May issue of the INTERNATIONAL DENTAL JOURNAL will be sent to some of the graduating classes of several dental colleges. This is a journal strictly devoted to professional interests, having no direct alliance with the ordinary business world. It is published by a company of dentists exclusively for dentists, and at present is the only distinct representative of the dental profession in the United States. A journal thus constituted should appeal to all graduates, as it constitutes in degree a post-graduate training in those ethical standards that was theirs to receive during their college life.

The sample numbers sent constitute, as it were, the advance thought that is supposed to lead more directly to the period of assured interest in dentistry as a daily practice. This only can come in months and years, but when it has arrived, the earnest practitioner avoids all dubious paths that lead beyond the borders of professional life.

The young men of this present graduating period, and those who succeed them from the colleges having longer courses, are cordially welcomed into the dental profession, and they can be assured there is room for all who have worthily labored through their preliminary training and are ready to meet all demands of the general public in moral excellence and practical ability.

